# Machine learning assisted Co_3_O_4_/NiO popsicle sticks-infused electrospun nanofibers for efficient oxygen evolution reaction

**DOI:** 10.1038/s41598-025-95130-7

**Published:** 2025-03-28

**Authors:** Azza A. Al-Ghamdi, Abdul Sami, Salah M. El-Bahy, Merfat M. Alsabban, Wajid Sajjad, Ahlam I. Al-Sulami, Muhammad Waseem Fazal, Reema H. Aldahiri, Fatimah Mohammad H. Al-Sulami, Muhammad Ali Khan, Naeem Akhtar

**Affiliations:** 1 College of Science, Department of Chemistry, University of Jeddah, 21589 Jeddah, Saudi Arabia; 2 Institute of Chemical Sciences, Bahauddin Zakariya University (BZU), Multan, 60800 Pakistan; 3 Department of Chemistry, Turabah University College, Taif University, P.O. Box 11099, 21944 Taif, Saudi Arabia

**Keywords:** Renewable energy, Oxygen evolution reaction, Water splitting, Co_3_O_4_/NiO popsicle sticks, Machine learning, Electrospinning, Energy, Chemistry, Energy science and technology, Materials science

## Abstract

Wide range of noble metal free bimetallic and trimetallic based electrocatalysts have been synthesized to develop efficient oxygen evolution reaction (OER) systems to-date, however, to determine which metal part of bimetallic and trimetallic electrocatalysts plays a significant role in controlling OER efficacy remains very challenging. To address this issue, herein we have employed machine learning (ML) for the first time to determine OER efficacy controlling metal element, thus leading to the development of an optimized bimetallic electrocatalyst. Briefly, we have designed a novel, simple ML optimized sustainable OER electrocatalyst based on Co_3_O_4_/NiO popsicle sticks (CNPS) infused polyaniline/cellulose acetate (a biopolymer) (PNCA) electrospun nanofibers supported on nickel foam (NF). ML optimized CNPS infused PNCA (CNPS@PNCA) electrode offers maximum and homogenous exposition of active sites and shows high OER activity by exhibiting low onset potential (1.41 V vs. RHE), overpotential (237 mV at 10 mA cm^−2^) and Tafel slope of 62.1 mV dec^−1^. Additionally, it shows a better stability of more than 100 h and is consistent with the reported literature.

**Supplementary Information:**

The online version contains supplementary material available at 10.1038/s41598-025-95130-7.

## Introduction

Energy plays a crucial part in the advancement of a nation’s economy, the quality of life, the improvement of infrastructure, and the facilitation of trade. There’s an imperative need for the implementation of novel reforms aimed at augmenting energy production and storage capacities to adequately address the escalating future energy requirements^1^. At present, fossil fuels are utilized as the predominant method to meet the increasing energy demands in diverse industrial sectors. Nevertheless, the unregulated and widespread utilization of fossil fuels has propelled our planet into a disconcerting condition characterized by a substantial escalation in worldwide temperatures and atmospheric contamination^2,3^. Additionally, it is imperative to explore global energy alternatives due to the finite nature of natural energy resources. Renewable energy sources, including wind, solar, and hydroelectric power, have been identified as viable alternatives to fossil fuels. However, their widespread implementation is sometimes impeded by many constraints, including limited supply based on geographical location and seasonal variations^4,5^. Consequently, there is a great interest in the investigation of alternative approaches and technologies that are more environmentally friendly, such as fuel cells, metal-air batteries, , and electrochemical water splitting to meet the growing need of energy^6^.

Electrocatalysis has gained significant attention in the advancement of renewable energy technologies, with the Oxygen Evolution Reaction (OER) occupying a prominent position in this transformational domain. The OER plays a crucial role as a half-reaction in diverse energy storage and conversion mechanisms, such as fuel cells, water electrolysis, and metal-air batteries^7,8^. Moreover, hydrogen is widely recognized as a progressively potent and ecologically sustainable energy resource in the near future. This can be attributed to its distinct benefits, including its elevated mass density, absence of carbon emissions, and remarkable purity levels^9^. Furthermore, it is widely acknowledged as an outstanding form of storable fuel and an energy carrier that possesses the capacity to produce an impressive 39.39 kWh kg^–1^ of electricity, thereby surpassing the energy density of diverse battery variants^10^. The OER plays a prominent role in the process of water splitting, however, the electrocatalytic properties of the OER are heavily dependent on the selection of materials. Noble electrocatalysts such as RuO_2_ and IrO_2_ are presently being recognized as the benchmark for OER^11,12^. However, the limited availability and significant expenses associated with them pose significant obstacles to their sustained economic feasibility^5,13,14^. Thus, the development of OER electrocatalysts that possess practicality, durability, non-toxicity, and excellent efficiency is of utmost importance. Substantial progress has been made in the improvement of electrocatalysts for OER within this particular framework. The emphasis has been placed on metals and their respective oxides, hydroxides, phosphides, and selenides^15,16^. It is worth mentioning that the mix of these catalysts can be tailored to modify their intrinsic characteristics, which collectively impact the OER activity^17^. Biomass conversion and water splitting are widely acknowledged as environmentally friendly approaches for the generation of hydrogen. The process of electrochemical water splitting presents a superior method for the acquisition of hydrogen, characterized by enhanced efficiency and productivity. Hence, this phenomenon has sparked the curiosity of researchers, prompting them to explore effective and economically viable electrocatalysts that can enhance the OER in both acidic and alkaline conditions.

In this context, it is important to acknowledge that the electrochemical stability of transition metal oxides is enhanced in an alkaline environment as opposed to an acidic one^18^. Transition metal oxides have garnered significant attention as electrocatalysts due to their high durability. Among these, cobalt-based oxides, including CoO, Co₃O₄, and MCo₂O₄ (where M represents Cu, Ni, Fe, etc.), have shown notable efficiency in promoting the OER under alkaline conditions^19^. Moreover, Bimetallic catalysts has emerged as promising materials as they offer significant advantages over monometallic catalysts due to their enhanced catalytic activity and stability, primarily driven by synergistic effects between the metal components. Zhang et al. reported that the interaction between two metals in CoNi-based bimetallic catalysts optimizes charge transfer and increases the number of active sites, leading to superior OER performance compared to monometallic systems^20^. Additionally, Barua et al. reported that bimetallic catalysts exhibit improved stability, as they are more resistant to degradation and corrosion under harsh reaction conditions, thereby extending their operational lifespan^21^. Thus, bimetallic Co_3_O_4_ and nickel oxide (NiO) have emerged as potential contenders for OER electrocatalysis owing to their inherent electrochemical characteristics, such as elevated conductivity and noteworthy catalytic efficacy. Moreover, the combined effect of their interaction inside a composite framework has the potential to result in improved electrocatalytic performance^22^. While bimetallic oxides exhibit considerable potential as catalysts, their catalytic performance often falls short of that achieved by noble metal catalysts, primarily due to their heterogeneous electrical conductivity and limited long-term durability^23^.

To tackle these issues, considerable attention has been given to nanofibers, owing to their remarkable characteristics such as a substantial surface area, elevated aspect ratios, effective charge conduction, customizable fiber diameters and porosity, straightforward manufacturing processes, potential for functionalization and most important their ability to maintain homogenous and uniform flow of ions at the electrode surface^24,25^. The utilization of these unique characteristics has made them widely employed in electrochemical applications to improve overpotential and stability, hence leading to enhanced performance in the OER. Recently, electrospun nanofibers have attracted greater attention in comparison to conventional fibers due to their adjustable porosity, which facilitates an increased quantity of active binding sites and improved interaction with ^−^OH ions^25–27^. In addition to electrospinning, solution blow spinning (SBS) has also emerged as an alternative to electrospinning for the fabrication of bulk nanofiber due to high production rate. While electrospinning allows precise control over fiber diameter and porosity, SBS provides a faster, cost-effective route for large-scale nanofiber synthesis without compromising structural integrity^28^. Moreover, the electrocatalytic activity of electrospun nanofibers can be significantly improved through surface modification with metal or metal oxide-based nanoparticles. Thus, the utilization of distinct multichannel electrospun nanofibers as a sacrificial template enables the fabrication of a high-performance OER electrocatalyst possessing well-defined hollow channels and a hierarchical pore structure^29^. This results in an increased number of active sites, thereby enhancing both the electron transfer rates and the stability of the designed electrocatalyst by offering homogenous and smooth flow of ions. Thus, the composition of electrospun nanofibers is of great significance in designing an efficient OER electrocatalyst with additional adsorption sites^30,31^.

Beyond the full-scale high-throughput experiments, ML-aided approaches have attracted increasing attention in predicting the governing factors that controls overpotential values for unknown compositions of OER electrocatalysts^5,32^. Predicting and optimizing the overpotential values for high-performance electrocatalysis is difficult because of the (1) varying physical behaviors of various metals^33^, (2) unique chemical reactivity^34^ and (3) difficulty to exactly determine affect the of metals in catalytic performance^32,35^. Furthermore, large variety of noble (Pt, Ir and Ru), non-noble metals (Ni, Fe and Co) and their alloys render the conventional experimental trial and error method, ineffective. On the other hand, ML as an AI subset, has revolutionized the modern world because it can analyze complex experimental data with high efficiency and determine the controlling metal element that affects overpotential along with making predictions of overpotential for unknown compositions^36,37^.

Considering the crucial factors, herein, we synthesized polyaniline (PN) and cellulose acetate (CA) modified electrospun nanofibers on a highly conductive nickel foam (NF) substrate with a porous sponge-like structure. We smartly selected PNCA as a material for nanofiber synthesis because of its tremendous features including exceptional electrical conductivity, little toxicity, a significant surface area, and a robust binding affinity through alternate imine and amine functional groups. CA was strategically chosen to improve the solubility, stiffness, and charge density of PN, thus facilitating the seamless fabrication of PNCA electrospun nanofibers with enhanced electron transport and adsorption properties. To get the maximum benefit of the PNCA electrospun nanofibers, their surface was further modified through the incorporation of Co_3_O_4_/NiO Popsicle sticks (CNPS). The incorporation of CNPS into the PNCA (CNPS@PNCA) electrospun nanofiber ensures maximum and uniform exposure of active sites, thereby enhancing the electrocatalytic performance. To get the best OER efficacy of the designed CNPS@PNCA electrode and to evaluate the impact of each constituent, various composition of CNPS (Ni(NO_3_)_2_·6H_2_Oand Co(NO_3_)_2_·6H2O (1, 1.5, 2 g) and impact of mass loading (0.5, 1, 1.5 mg) were optimized in terms of overpotential values by using ML models. Results reveals that the ML optimized designed OER electrocatalysts demonstrates, a higher adsorption capacity and exceptional long-term stability which can be attributed to the, (i) ML optimized ratio of CNPS, (ii) seamless electron transport facilitated by the smooth and uniform structure of the PNCA, and (iii) strong cohesive interface interaction between PNCA and CNPS along with robust hydrogen bonding between the hydroxyl groups of CA and the secondary amines of PN. The designed ML optimized CNPS@PNCA electrode shows remarkable electrocatalytic activity for the OER, characterized by a lower onset potential (1.40 V vs. the reversible hydrogen electrode, RHE) and a lower Tafel slope of 62.1 mV dec^−1^ in comparison to standard electrocatalysts. The observed improvement in performance can be attributed to the synergistic effects that arise from the combination of CNPS and PNCA electrospun nanofibers. This combination results in better stability and durability over a period of 24 h, as seen by the findings from chronoamperometric experiments.

## Results and discussion

### Morphological analysis of the synthesized material

**Fig. 1:**
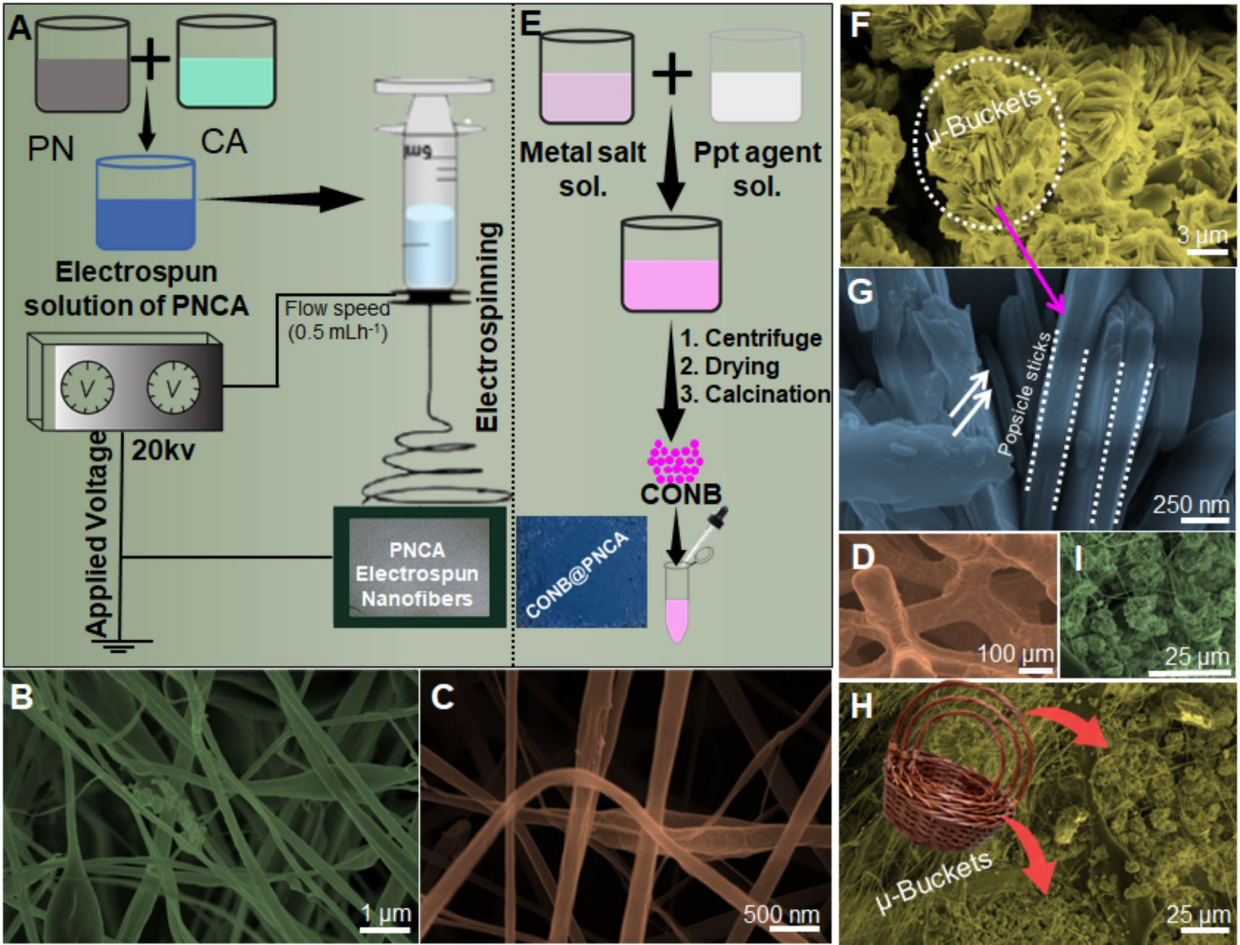
Shows the synthesis roadmap for PNCA-based electrospun nanofibers on NF (**A**). Image (**B** & **C**) shows the SEM images of PNCA nanofibers. Panel (**E**) depicts the CNPS nanocomposite synthesis and its drop-casting on PNCA, with an inset image. While (**F** and **G**) show low- and high-magnification SEM images of the CNPS nanocomposite, (**H** and **I**) illustrate the SEM images of CNPS@PNCA.

**Fig. 2:**
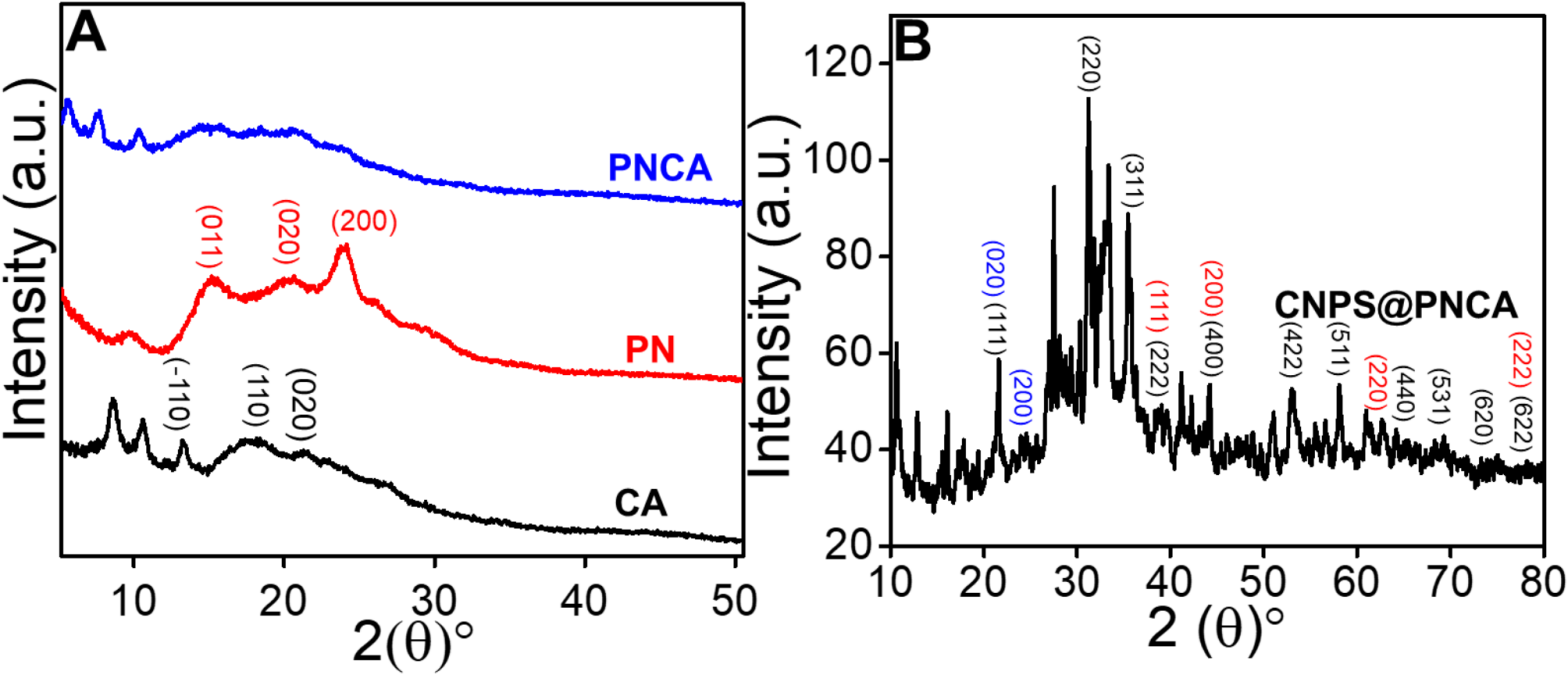
(**A**) Representing XRD spectra of CA, PN, and PNCA and (**B**) shows the XRD spectra of CNPS@PNCA.

Scanning Electron Microscope (SEM) was carried out to study the surface morphology of CNPS and infusion of CNPS onto the PNCA electrospun nanofibers (Fig. 1). Figure 1A illustrates the roadmap for the fabrication of PNCA-based electrospun nanofibers on the surface of NF. Low- and high-resolution SEM images (Fig. 1B,C) clearly show the formation of homogeneous electrospun nanofibers at NF. As evident from SEM, majority of the nanofibers are smooth and free of beads, exhibiting ribbon-like morphology with a size range of 40–50 nm. Figure 2D presents the SEM image of NF with a sponge-like structure. Literature has shown that NF, when coated with homogeneous PNCA electrospun nanofibers, provides excellent accessibility, facilitating rapid and uniform electron and ion movement across the electrode surface^38^. Figure 1E illustrates the various steps involved in the synthesis of CNPS, along with a digital image showcasing the surface morphology of the PNCA electrospun nanofibers. Furthermore, the SEM images captured at lower and higher magnifications (Figure F & G), demonstrates the presence of popsicle sticks shaped CNPS nanocomposite. These nanosized popsicle sticks possessed a diameter with size in the range of 70–90 nm. These popsicle sticks further arranged in such a way that they form the shape of a µ-buckets. The infusion of CNPS onto the surface of PNCA is clearly depicted in Fig. 1H,I.

### Compositional analysis of the synthesized material

X-ray diffraction (XRD) analysis has been employed to evaluate the crystallinity and phase purity of the synthesized materials. XRD spectra of PN indicate a lack of well-defined, narrow peaks, indicating a very low degree of crystallinity (Fig. 2 A (red line)). This can be attributed to the intrinsic structural disorder within the polymeric matrix of PN. In more detailed way, it displays a wide reflection peak at around 26.16°, which corresponds to the crystallographic plane (200). Furthermore, it shows large reflection peaks at around 15.18 and 20.62°, indicating the existence of crystal planes (011) and (020). CA exhibited distinct XRD pattern throughout the angular range of 10 to 25° (Fig. 2A (black line)). The presence of a prominent peak at approximately 20° indicates the amorphous characteristics of CA. Additionally, the peaks at angles of 13.22, 17.72, and 21.3° can be attributed to the crystal diffraction planes (1̅10), (110), and (200), respectively. The presence of newly formed peaks in the XRD pattern indicate the creation of PNCA nanofibers (Fig. 2A (blue line)), and in accordance with the reported literature^39^. The observed structural alteration can be ascribed to the creation of weak hydrogen bonds between the ^−^OH groups of polar CA and the R_2_NH moieties of the electron acceptor, PN. It is worth mentioning that PN is widely recognized for its significant electron-accepting characteristics. Consequently, polar CA demonstrates π-orbital interactions with the amines of PN. The findings suggest that the composite contains three distinct crystalline phases due to the inherent crystalline properties of CA. These phases consist of the mobile amorphous fraction, the portion of CA chains that bind to the dispersed PN (termed the rigid amorphous fraction), and the fraction of CA located on the outer surface of the electrospun nanofibers. The presence of distinct peaks at angles less than 10° is ascribed to the phenomenon of crystallization of CA^40^. This crystallization process is triggered when the CA solution mixture is subjected to increased stretching during the electrospinning process, hence promoting the development of crystals to a greater extent^41^. On the other hand, the wide peaks seen in the range of 10 to 30° suggest the existence of parallel and perpendicular periodicity in the PN chains^42^. The presence of a prominent peak at around 21° is indicative of the crystalline nature of the PN and in accordance with the reported literature^43^. Additionally, the crystalline nature of the synthesized CNPS was investigated using XRD (Fig. 2B), and the results revealed the presence of distinct diffraction planes of NiO (labeled with red color), namely (111), (200), (220), and (222), with corresponding diffraction angles of 38.7, 44.2, 61.4, and 78.7, which matched well with JCPDS no. 01–078-0643. While peaks at 21.5, 31.2, 35.5, 38.6, 44.2, 54.5, 58.9, 64.6, 69.1, 74.7 and 78.6° indexed as (111), (220), (311), (222), (400), (422), (511), (440), (531), (620), and (622), which matches well with Co_3_O_4_ (labeled with black color) and JCPDS no. 01–076-1802. Furthermore, the XRD analysis identified characteristic peaks for the nanocomposite of PNCA (labeled with blue color), specifically at 21.6° and 24.5°, corresponding to the (020) and (200) crystallographic planes, respectively.

The FTIR spectra of PNCA electrospun nanofibers exhibit characteristic peaks that can be attributed to CA and PN, as depicted in section S1 (Figure S1). The distinct peaks at 1733 and 1369 cm^−1^ indicates the occurrence of the C=O stretching and C–CH_3_ bending vibrational modes of CA^44^, respectively. In addition, observable adsorption bands corresponding to the C–O and C–O–C (glycosidic linkage)^38,45^ are evident at wavenumbers of 1219 and 1033 cm^−1^, respectively. The presence of a quinonoid (N=Q=N) and benzenoid (N–B–N) doublet at 1583 and 1492 cm^−1^^26,46^, respectively, indicates the presence of the conductive form of PN in the characteristic fingerprint area. Concurrently, the observed prominent peak at a wavenumber of 1733 cm^−1^ corresponds to the vibrational stretching of the carbon–oxygen double bond (C=O) in CA. Significantly, variations in the magnitude of the PN peaks function as a discernible indicator for the PNCA composite formation. Additionally, the presence of the C–H band at 730 cm^−1^ in the PNCA composite further supports its successful formation. In addition, the identification of C–N stretching vibrations occurring at a wavenumber of 1306 cm^−1^ indicates the presence of π-electron delocalization inside the composite structure^45,47^. The peaks in the 600 to 700 cm⁻^1^ region indicate unsaturation in the benzene ring, attributed to out-of-plane bending vibrations of C–H bonds in the 1,4-disubstituted benzenoid rings. Additionally, hydrogen bonding is facilitated between the –OH groups of polar CA and the secondary amines of the electron-accepting PN. This is indicated by the observed intensity of the secondary amine adsorption band in PN molecules, which is typically found at approximately 3245 cm^−1^. The conductive character of PN is indicated by a significant and wide absorption band observed in its spectra, which spans around 2000 cm^−1^. The emergence of this unique band can be attributed to the phenomenon of charge barrier absorption occurring within the doped polymer. The spectrum analysis reveals the presence of supplementary spectral characteristics, specifically peaks falling within the spectral range of 700–740 cm^−1^. These peaks are indicative of the bending vibrations associated with the C–H bonds. Similarly, the occurrence of peaks ranging from 801 to 824 cm^−1^ provides evidence for the existence of carbon–carbon double bonds. The presence of the characteristic peaks corresponding to the quinonoid and benzenoid doublet provides more evidence for the creation of the emeraldine base state of PN. It is worth mentioning that a distinct peak observed at around 3245 cm^−1^ signifies the presence of N–H stretching vibrations in secondary amines^26,48^. Furthermore, asymmetric C-H stretching vibration has been observed at 3032 cm^−1^. Figure S2 illustrates the Raman spectra of CA and PN (Section S1). In the PN spectrum, two discernible peaks corresponding to bipolarons have been observed: the C–N^+^, N=C, and C=C stretching vibration have been observed 1348, 1487 and 1572 cm^−1^, respectively^49^. Moreover, band at 955 cm^−1^ can be attributed to C-H out of plane bending mode. In case of CA, distinct Raman bands at 1772 and 1430 cm^−1^ correspond to carbonyl (C=O) groups and the CH_3_ groups (deformation vibrations) stretching vibrations in acetyl moieties, respectively^50^. Similarly, band at 1320 cm^−1^ can be attributed to CH_3_ group vibrations^51^.

### Prediction of overpotential using ML on experimental dataset (ED)

To design highly efficient electrocatalysts with low overpotential, various ML regression models were employed to establish feature-objective relationships and predict optimal synthesis conditions. Given the relative ease of experimentally determining overpotential, the scope of this study was expanded to leverage ML for predicting overpotential by optimizing catalyst structure, and elucidating catalyst behavior during reactions. Different ML regression models including decision tree regression (DTR), linear regression (LR), support vector regression (SVR), gradient boosting regression (GBR), random forest regression (RFR), and K-nearest neighbor regression (KNNR) were employed on the ED relating various composition of CNPS (Ni(NO_3_)_2_·6H_2_O (0.5, 0.6, 0.7, 0.8, 0.9, 1.1, 1.2, 1.3, 1.4, 1.5, 1.6, 1.8, 1.9, 2 g), and Co(NO_3_)_2_·6H_2_O (1, 1.5, 2 g) and mass loading (0.5, 1, 1.5 mg) with their corresponding overpotential values. These algorithms were trained and tested using a dataset split into 70% for training, 15% for validation, and 15% for testing. Statistical measures such as root mean square error (RMSE) and the coefficient of regression (R^2^) were used to evaluate their predictive accuracy, with the optimal model characterized by the lowest RMSE and the highest R^2^. In addition to predicting overpotential, the models were employed to optimize synthesis conditions and provide insights into material and structural parameters critical to enhancing electrocatalytic performance.

**Fig. 3:**
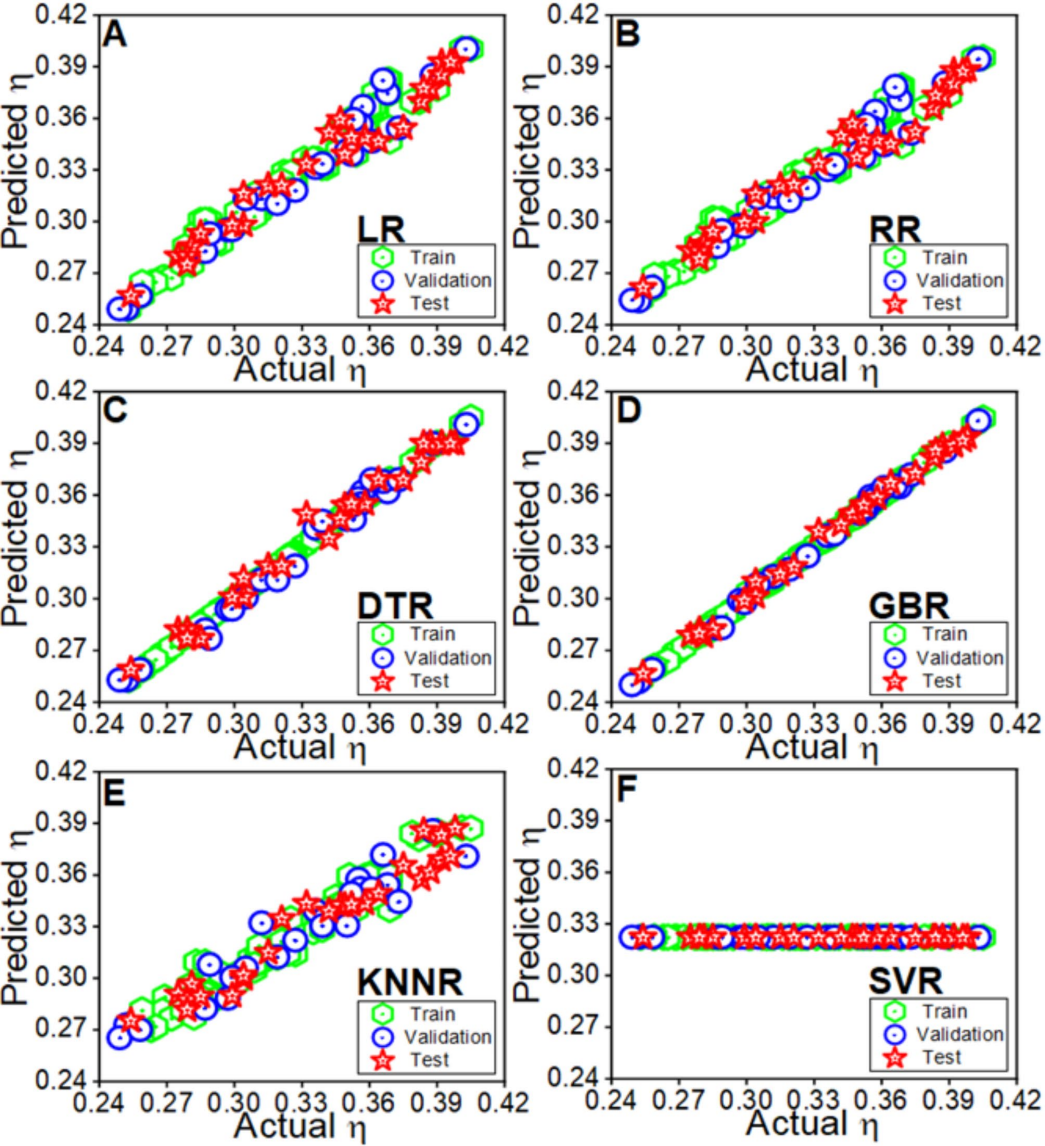
The comparative analysis of prediction of overpotential response, employing eight distinct regression model including (**A**) LR, (**B**) RR, (**C**) DTR, (**D**) GBR, (**E**) KNNR and (**F**) SVR, respectively.

**Fig. 4:**
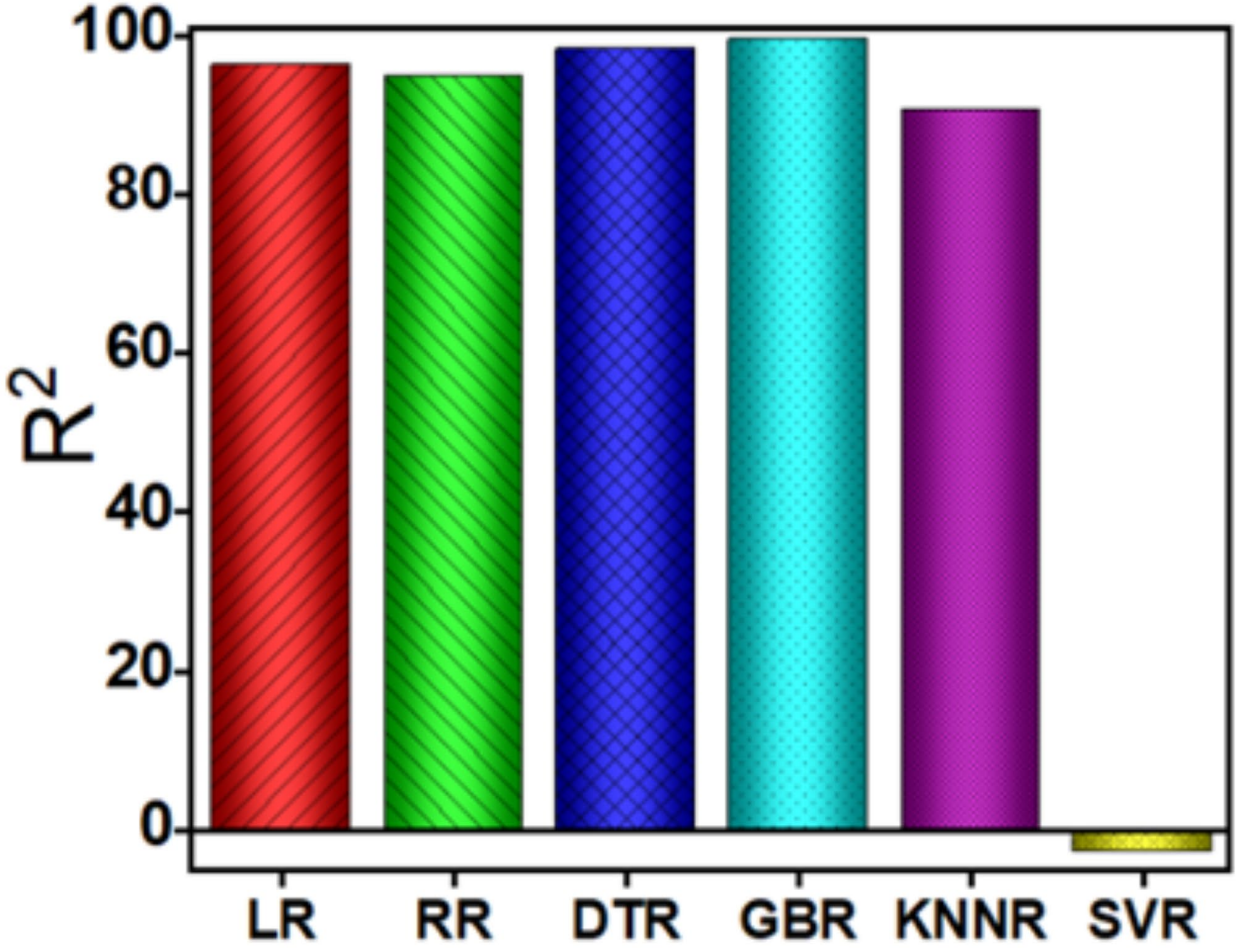
The coefficient of regression (R^2^) of different regression models.

Figure 3 showcases the predictive performance of different algorithms, with LR (A), DTR (C), and GBR (D), demonstrating notable correlations between actual and predicted overpotential values, while SVR exhibits the least efficacy. The linearity of the data points in these scatter plots indicates the models’ ability to capture the underlying relationship between the input features and overpotential. Models such as GBR, LR, and DTR exhibit a near-perfect alignment of predicted and actual values, reflecting strong predictive capabilities with minimal scatter. Conversely, SVR shows significant scattering, indicative of poor model generalization and weak correlation between predicted and actual values, as further confirmed by its negative R^2^ value. Comparative R^2^ values for LR, RR, DTR, GBR, KNNR, and SVR were calculated as 96.23, 94.7, 98.29, 99.54, 90.61, and − 2.817%, respectively (Fig. 4). The corresponding RMSE values are summarized in Table S1, highlighting GBR as the most effective model, followed closely by XGBR and RFR. These findings underscore the reliability of the ML model in predicting overpotential, demonstrating its robustness and broad applicability. These results validate the model’s predictive efficacy in generalizing diverse scenarios. Additionally, Figure S3 in Section S2 highlights the relative importance of the features used in training the LR model, offering valuable insights into the key factors that influence overpotential.

### Electrocatalytic behavior of the designed electrode

**Fig. 5:**
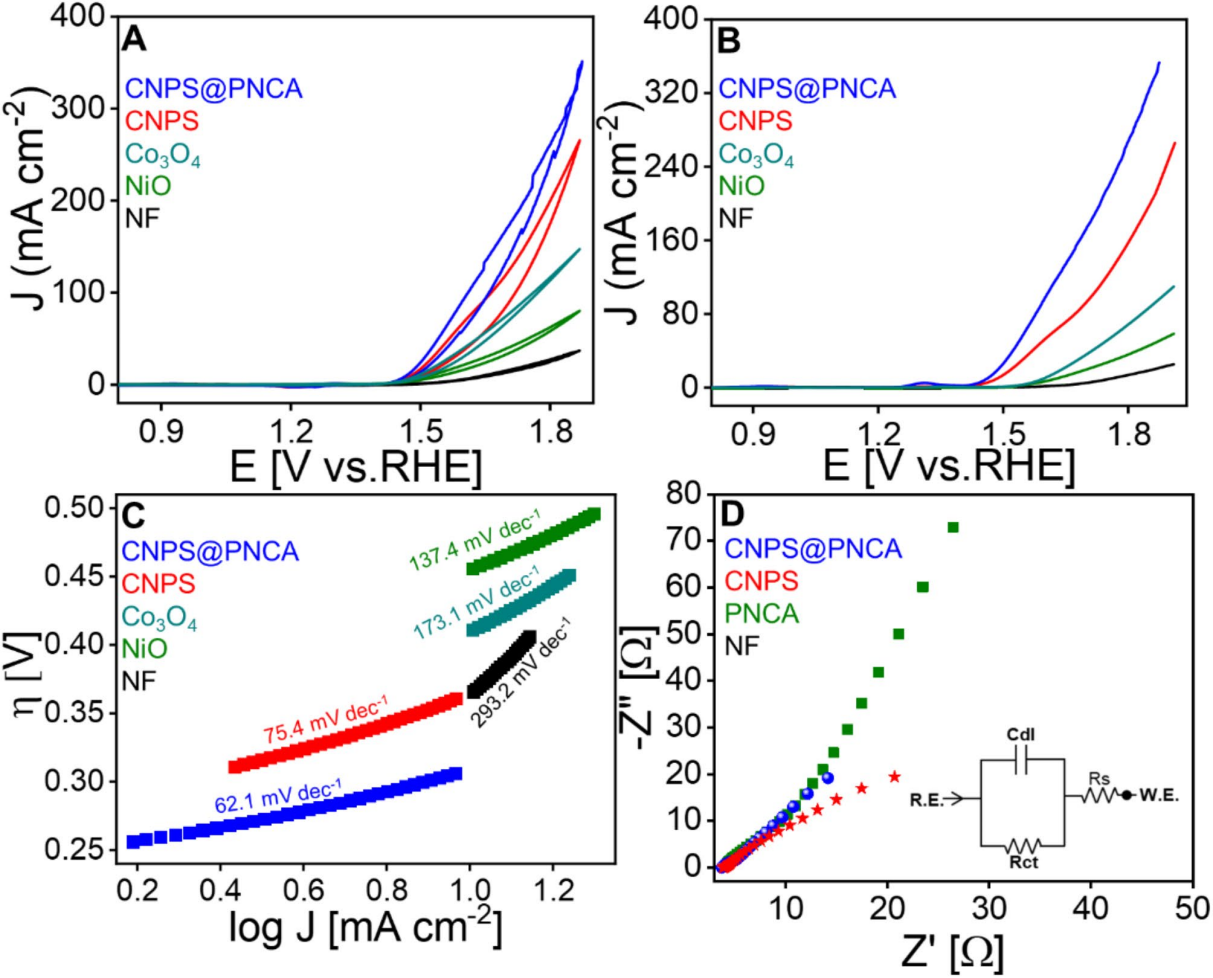
Electrocatalysis measurements of CNPS@PNCA and CNPS (**A**) LSV (**B**) CV (**C**) Tafel plot of CNPS@PNCA and for CNPS obtained from the CV in 1 M KOH and (**D**) EIS Nyquist plot with the Randle’s circuit.

The electrocatalytic behavior of the all the fabricated materials (CNPS@PNCA, CNPS, NiO, Co_3_O_4_, NF) were assessed using cyclic voltammetry (CV) and linear sweep voltammetry (LSV) techniques. A three-electrode system was employed, consisting of an Ag/AgCl electrode, Pt wire and modified electrode, as the reference, counter, and working electrodes, respectively. The onset potential and overpotential of all the fabricated materials were determined from CV and LSV. CV (Fig. 5A, S4A-E) and LSV (Fig. 5B, S4F) were performed within a potential window of 0.85–1.85 V vs. reversible hydrogen electrode (RHE), using a scan rate in the range of 5–25 mV s^−1^ in a 1 M KOH electrolyte solution. The OER reaction was observed through the formation of oxygen bubbles on the surface of the electrode. As evident from CV and LSV, CNPS@PNCA based composite has shown low onset potential (1.42 V), as compared to CNPS (1.42 V), NiO (1.47 V), Co_3_O_4_ (1.56 V), and NF (1.59 V) at 0.1 mA cm^−2^ current density^52,53^. Similarly, CNPS@PNCA has shown low overpotential (237 mV vs. RHE) as compared to CNPS (249 mV vs. RHE), NiO (380 mV), Co_3_O_4_ (430 mV), and NF (530 mV) at current density (10 mA cm^−2^). To ensure the reproducibility and reliability of the results, CV and LSV measurements were performed in triplicate for all the designed materials as shown in Figure S4 E & F respectively. Furthermore, we have evaluated the reaction kinetics and intrinsic electrocatalytic activity of CNPS@PNCA, CNPS, Co_3_O_4_, NiO, and NF electrodes by deriving the Tafel slope from LSV and plotting a graph of the logarithm of current density (log j) against overpotential (Fig. 5C). The results indicated that CNPS@PNCA exhibited a significantly lower Tafel slope of 62.1 mV dec^−1^ as compared to CNPS’s (75.4 mV dec^−1^), NiO (137.4 mA cm^−2^), Co_3_O_4_ (173.1 mA cm^−2^), and NF (293.1 mA cm^−2^) thus suggesting a higher reaction rate and a more favorable water oxidation mechanism with enhanced OER intrinsic kinetics. The calculated Tafel slope is also in good competition with the already reported literature as well (Table S3). Furthermore, CNPS@PNCA exhibited the highest current density of 120.04 mA cm^−2^, significantly outperforming CNPS (61.2 mA cm^−2^), Co3O4 (15.37 m Acm^−2^), NiO (9.1 mA cm^−2^), and NF (1.68 mA cm^−2^) at an overpotential of 400 mV (1.63 V vs. RHE) demonstrating its higher electrochemical performance^54^. Additionally, the electrochemical impedance spectroscopy (EIS) was next employed to assess the charge transfer resistance (Rct) associated with the OER, as depicted in Fig. 5D and S5, S6 in section S3. The EIS data was analyzed using the Randles circuit models, comprising solution resistance (Rs), double-layer capacitance (Cdl), Warburg impedance (Zw) and Rct. Rs represents the ionic resistance of the electrolyte, Cdl models charge storage at the electrode–electrolyte interface, Zw models the diffusion of species to the electrode surface and Rct denotes the resistance to electron transfer. Figure CNPS@PNCA exhibited a low Rct of 9.06, as compared to CNPS (14.97), Co_3_O_4_ (13.87), NiO (26.04), NF (45.19) without the polymer additives and only PNCA (23.46) respectively (Fig. 5D and S5). The reduction in Rct from 14.79 to 9.06 Ω, representing a 38.7% decrease, is significant. Furthermore, the presence of two semicircles in the Nyquist plot of the NF suggests that the electrochemical process is diffusion-controlled. This substantial improvement in Rct highlights improved electron transfer kinetics, attributed to the PNCA polymer resulting in forming conductive channels, enhancing surface area, facilitating electron shuttling, and strengthening interactions with metal oxides. These features suggest efficient electron transfer, a conductive nature of the electrocatalyst, and the occurrence of simple catalytic processes on the electrode surface. The determination of the minimum value of *Rct* for CNPS@PNCA is significant, as it signifies a superior electronic transfer capability. This enhanced performance of CNPS@PNCA electrode can be attributed to its strong adsorption capacity of OH^−^ ions, which in turn facilitates smooth and fast OER activity. The strong adsorption capability of CNPS@PNCA could be attributed to its maximum and smooth exposition capability of active sites due to, (i) bucket shaped architectures of CNPS with nanosized popsicle sticks, (ii) bimetallic synergistic effect, and (iii) presence of homogenous PNCA nanofibers over the entire surface.

**Fig. 6:**
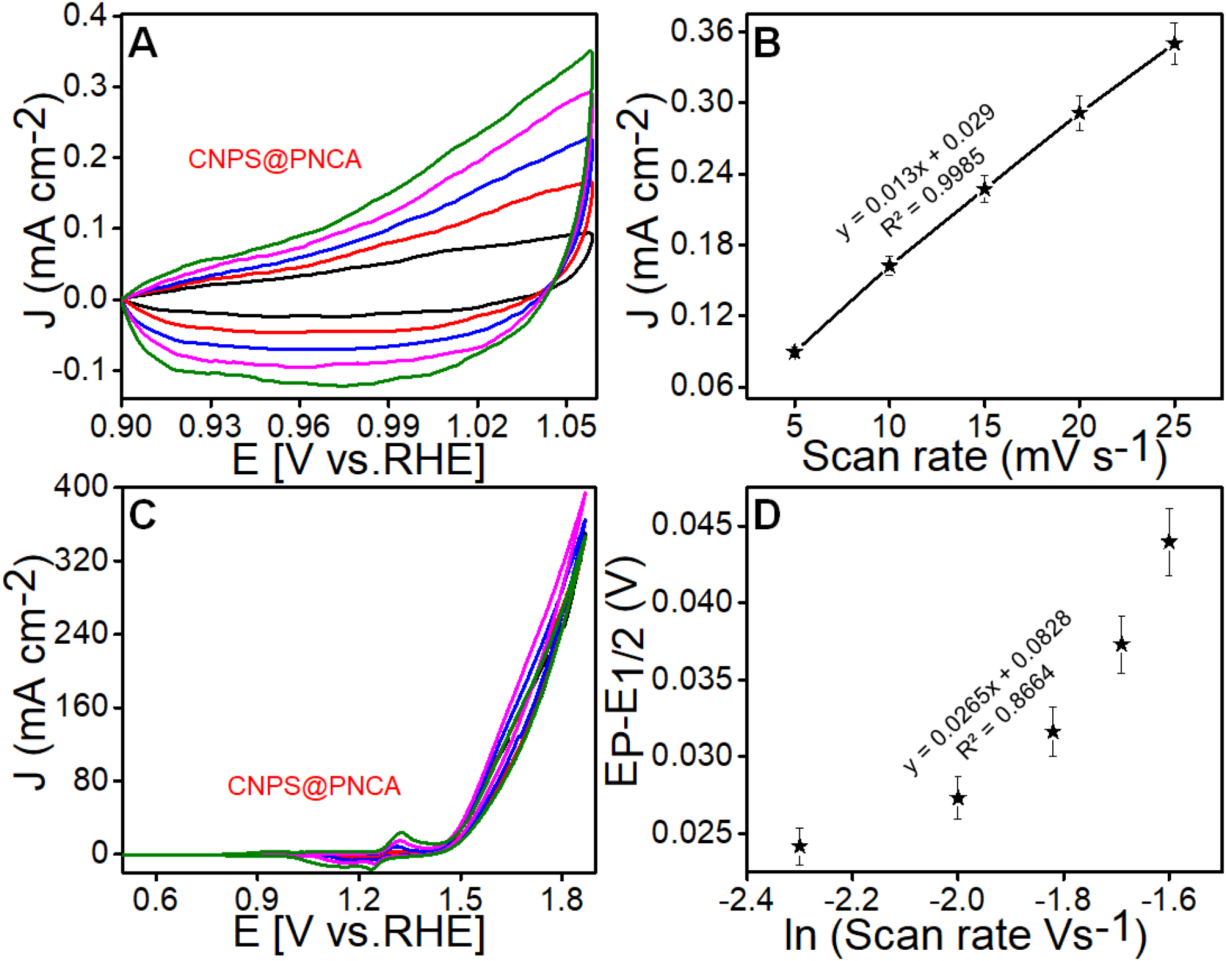
(**A**) Non-faradaic region CV of CNPS@PNCA (**B**) graph plotted between j and scan rate. (**C**) CV of CNPS@PNCA with scan rates from 5 to 25 mV s^−1^ (**D**) plot between redox peak potential and logarithm of scan rate.

Further, electrochemically active surface area of the designed CNPS@PNCA electrode, we measured the double-layer capacitance (Cdl). Thus, CV was performed in non-faradaic region from 0.9 to 1.05 V vs RHE at scan rates of 5–25 mV s^−1^ in 1.0 M KOH electrolyte solution (Fig. 6A). From the CV curves, plot of current density versus scan rate was derived and shown in Fig. 6B. The Cdl was calculated by using Eq. S4 and its value was found to be 6.5 mF cm^−2^. Further, the electrochemically active surface area was calculated by dividing the Cdl (6.5 mF cm^−2^) with specific capacitance (0.04 cm^2^) by applying Eq. S4. The calculated electrochemically active surface area of 162.5 cm^2^ is observed at the surface of CNPS@PNCA, thus, suggesting the existence of wide range of electroactive sites, and is consistent with the already reported literature (Table S2).

####  Investigating reaction rates and mechanisms

Further, the efficacy of designed electrode in terms of OH^−^ ion adsorption capability was calculated through adsorption coefficient (ks) values by applying the Laviron equation. Briefly, CV was performed in 1 M KOH electrolyte at increasing scan rates of 5 to 50 mV s^−1^ (Fig. 6C). The results obtained from CV indicated a positive correlation between the scan rate value and the magnitude of redox peaks. This relationship led to the occurrence of pre-oxidation at the surface of the modified electrode. Further, a calibration graph between log of scan rate versus potential was derived from the Fig. 6C. Results (Fig. 6D) reveals a positive and linear shift. By putting all the value in Eq. S7, the value of ks was determined and it was found to be 0.48 s^−1^, which suggest a significant binding effectiveness of CNPS to the OH- intermediate across the complete surface area of our designed CNPS@PNCA electrode.

Next, we examined the OER mechanism at the surface of our designed CNPS@PNCA electrode. Briefly, the OER involves the formation of oxo intermediates and proceeds through a four-step proton-electron transfer mechanism, Eqs. (1) to (4).

1 $${\text{M }} + {\text{ OH}}^{ - } \leftrightarrow {\text{ M}} - {\text{OH }} + {\text{ e}}^{ - }$$

2 $${\text{M}} - {\text{OH}} - \, + {\text{ OH}}^{ - } \leftrightarrow {\text{ M}} - {\text{O }} + {\text{ H}}_{{2}} {\text{O}} + {\text{e}}^{ - }$$

3 $${\text{M}} - {\text{O }} + {\text{ OH}}^{ - } \leftrightarrow {\text{ M}} - {\text{OOH }} + {\text{ e}}^{ - }$$

4 $${\text{M}} - {\text{OOH }} + {\text{ OH}}^{ - } \leftrightarrow {\text{ M }} + {\text{ O}}_{{2}} + {\text{ H}}_{{2}} {\text{O }} + {\text{e}}^{ - }$$

It is assumed that the CNPS@PNCA electrode experiences a change in its electronic structures due to the oxidation of Co^2+^ to Co^3+^ and Ni^2+^ to Ni^3+^. This process leads to the creation of active species for the OER, such as Co^3+^-(OH) hydroxocobalt, and Ni^3+^^−^(OH) hydroxonickel intermediates. These intermediates are further coupled with deprotonation and subsequently form peroxide species, namely oxy hydroxocobalt Co^3+^-O(OH) and oxy hydroxonickel Ni^3+^-O(OH) intermediates. The intermediate formed thereafter undergoes additional interaction with the adsorbed hydroxide (OH^−^) species, leading to the formation of dioxygen (O_2_) molecules on the surface of the developed CNPS@PNCA electrode (Scheme 1) and similar kind of mechanism is also reported in the literature^26^. The rapid and efficient production of O_2_ is facilitated by a densely interconnected network of PNCA electrospun nanofibers. Briefly, PNCA electrospun nanofibers enable a uniform distribution of ions across the entire surface and maximizes the exposure active sites of bucket shaped architectures of CNPS with nanosized popsicle sticks over the entire surface. Consequently, the designed electrode exhibits a high capacity for adsorbing OH^−^ ions, owing to the strong binding affinity between the CNPS and the OH^−^ intermediates.

**Fig. 7:**
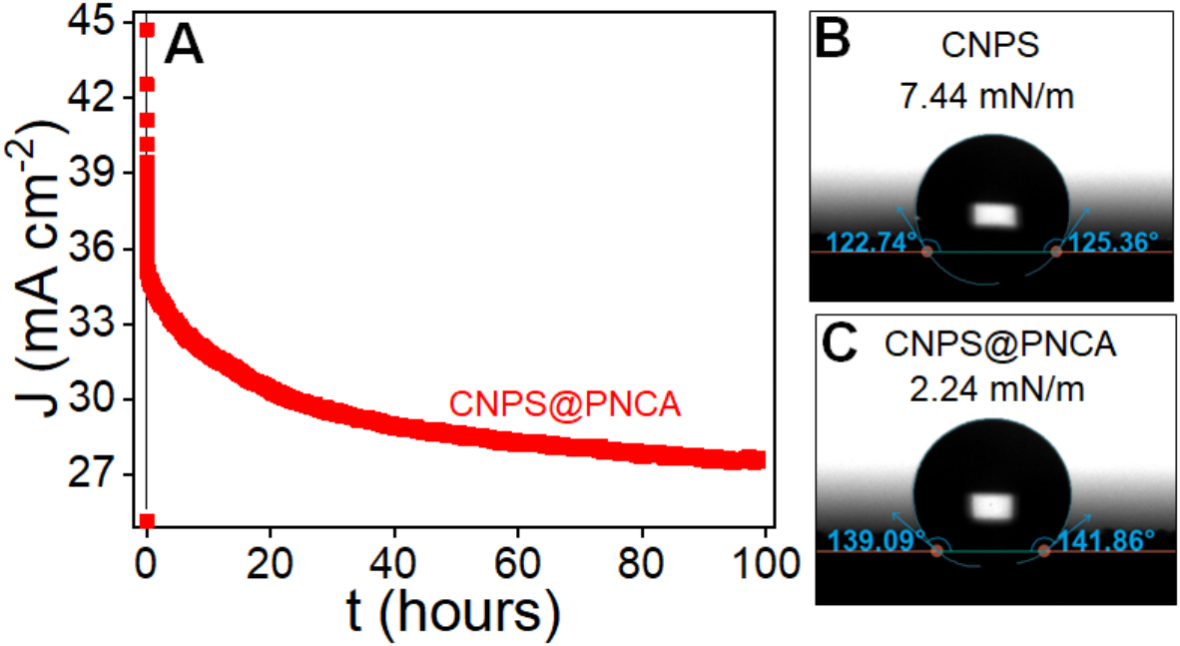
(**A**) Chronoamperometric measurement to evaluate stability and durability. Contact angle with surface energies of (**B**) CNPS and (**C**) CNPS@PNCA.

To evaluate the electrocatalyst efficacy, electrochemical stability is another important key element. Thus, chronoamperometric measurements were conducted to evaluate the durability and stability of our designed CNPS@PNCA electrode at a potential of 1.5 V vs RHE in 1 M KOH solution for a duration exceeding 100 h (Fig. 7A). The experimental findings indicate that the designed electrode exhibited an initial current density of 35 mA cm^−2^, which decreased to approximately 27 mA cm^−2^ after 100 h, representing a 22% deterioration. The enhanced stability was further supported through contact angle measurements by observing the binding energies at the surface of designed electrodes (Fig. 7B,C). Results reveals that the designed CNPS@PNCA electrode shows a low binding energy (2.24 mN m^−1^) compared to CNPS (7.44 mN m^−1^), thus leading to a favorable OER for water splitting. A reduction in binding energy of CNPS@PNCA compared to CNPS could be attributed to the robust cohesive interface interaction between PNCA and CNPS popsicle sticks, and these results are in accordance with the reported literature. After stability test, SEM analysis was carried out to investigate the morphological features of the CNPS. SEM images (low and high magnified), obtained after chronoamperometric measurements (Figure S7), revealed the formation of µ-buckets with interconnected popsicle sticks with insignificant structural degradation, likely due to prolonged electrochemical stress and surface reconstruction processes. It is noteworthy that the enhanced performance in terms of over potential, and Tafel Slope of our designed electrode exhibits significant competitiveness and in certain cases even surpasses the majority of metals oxide-based electrocatalysts (Table S3).

## Conclusion

Herein, ML optimized CNPS coated PNCA electrospun nanofiber based electrocatalyst was synthesized on the surface of NF. A data-driven ML approach was employed to optimize the material composition, predict and validate the material electrocatalytic efficacy. ML optimized CNPS@PNCA nanocomposite was characterized by SEM, FTIR, XRD, Raman spectroscopy and contact angle. Whereas OER electrocatalytic efficacy of the designed electrode was evaluated by various characterization techniques such LSV, CV, EIS and chronoamperometry. ML optimized CNPS@PNCA nanocomposite has shown high electrocatalytic activity by exhibiting low onset potential (1.40 V) as compared to CNPS (1.41 V), NiO (1.45 V), Co_3_O_4_ (1.55 V), and NF (1.56 V). This improved electrocatalytic performance and high stability in alkaline condition can be ascribed to optimized synergistic effects of CNPS and their infusion with conductive nature of PNCA electrospun nanofibers, which provides maximum and homogenous exposition of active sites, smooth adsorption of OH^−^ ions and long-term cycle stability, thus resulting in enhanced electrocatalytic performance.

## Methods

The chemicals and solvents employed in the synthesis of electrode and subsequent electrochemical analysis were of the highest purity, adhering to the requirements set for analytical grade materials. The reagents were utilized without the requirement of additional purification procedures. Cellulose acetate (CA) with a molecular weight of 100,000 was obtained from Acros Organics. Sodium hydroxide, acetone (with a purity of 99%), and hydrochloric acid (with a concentration of 37% and classified as extremely pure) were purchased from Sigma Aldrich. The chemicals including aniline with a purity of 99.5%, N, N-dimethylacetamide (DMAc) with a purity of 99.9%, nickel nitrate, and cobalt (II) nitrate hexahydrate were procured from Alfa Aesar. Furthermore, ammonia solution, ammonium persulfate, and three-dimensional NF, were obtained from Duksan Reagents.

### Synthesis of CNPS@PNCA electrospun nanofibers

The process for the synthesis of CNPS@PNCA electrospun nanofibers comprises of four sequential stages as follows:

### Synthesis of Co_3_O_4_

For the fabrication of Co_3_O_4_, Co(NO_3_)_2_·6H_2_O solution was prepared in distilled water and stirred (200 rpm) at 25 °C for 30 min. Subsequently, NH_3_ solution was added drop wise into the continuously stirred solution. After 6 h of additional stirring, the solution was centrifuged and washed with DI and ethyl alcohol. The resulting precipitates were dried in an oven at 80 °C for 6 h and calcined in a furnace at 450 °C for 2 h.

### Synthesis of NiO

Similarly for NiO, Ni(NO_3_)_2_·6H_2_O) solution was prepared in distilled water and allowed to stir (200 rpm) for 30 min at 25 °C. Then NH_3_ solution was added drop wise as a precipitating agent_._ The solution was stirred for 6 h, followed by centrifugation and washing. The resulting precipitates were dried in an oven at 80 °C for 6 h and calcined in a furnace at 450 °C for 2 h.

### Synthesis of Co_3_O_4_/NiO popsicle sticks (CNPS)

Multiple solutions were made by using varying amounts of Ni(NO_3_)_2_·6H_2_O (1, 1.5, 2 g) and Co(NO_3_)_2_·6H_2_O (0.5, 0.6, 0.7, 0.8, 0.9, 1, 1.1, 1.2, 1.3, 1.4, 1.5, 1.6, 1.7, 1.8, 1.9, 2 g) in distilled water with continuous stirring (200 rpm) at 25 °C for obtaining the material with optimum overpotential value. Following this, a solution composed of water and the precipitating agent (NH_3_ precursor) was carefully introduced in a controlled manner, drop by drop, to the continuously stirred solutions. Subsequently, the precipitates obtained were subjected to a series of centrifugation processes, wherein they were washed sequentially with DI and ethyl alcohol. The resultant product was dried in an oven at 80 °C for 6 h and later exposed to a calcination procedure in a furnace, where a temperature of 450 °C was maintained for 2 h. This process led to the formation of various bimetallic oxides from which the material having equimolar concentration of these salts was further studied due to low overpotential value.

### Synthesis of polyaniline (PN)

The synthesis of PN was conducted using oxidative polymerization at ambient temperature, employing a procedure that had been previously reported. Briefly, a solution of hydrochloric acid (HCl) with reduced concentration was added to a sample of aniline (0.22 mol) and agitated for a duration of 30 min while being kept in an ice bath, ensuring that the temperature remained below 5 °C during the entire procedure. Following this, the polymerization agent, namely ammonium persulfate, which had been previously produced as a solution with a concentration of 0.11 M in DI water, was introduced into the aniline solution in a controlled manner, with a flow rate of approximately 60 mL per hour. The solution obtained was subsequently agitated for a duration of 12 h, resulting in the attainment of complete polymerization and the production of a suspension with a dark green coloration. The PN product doped with acid was separated using paper filtration and subsequently rinsed with deionized water until the filtrate achieved a neutral pH. The remaining filtration product was mixed with 100 mL of concentrated ammonia solution (NH_3_), followed by an additional 24-h period of continuous stirring. The precipitates underwent a thorough washing process and were subsequently subjected to vacuum conditions in an oven set at a temperature of 60 °C for drying.

### Synthesis of PNCA electrospun nanofibers

A solution with a concentration of 20 weight/volume percent of CA was meticulously created by dissolving CA in a binary solvent system comprising of DMA and acetone, while carefully preserving a precise volumetric ratio of 2:1. The solution underwent continuous magnetic swirling on a hot plate at room temperature until it transformed into a transparent and uniformly homogeneous mixture. Following that, a precise 1% concentration of PN was carefully dissolved in the CA solution and subsequently treated with ultrasonication for a period of 30 min to guarantee thorough uniformity. To account for environmental factors, the electrospinning was carried out under controlled temperature (25 °C) and relative humidity (40%) to ensure consistent fiber morphology. The resultant solution was later transferred into a syringe to initiate the process of fiber production. The electrospinning procedure was conducted using a 20-kV potential, with a controlled flow rate of 0.5 mL h^−1^, while maintaining a constant distance of roughly 12 cm between the collector (i.e., nickel foam) and the needle tip (with a gauge size of 20). As a result of these, we successful fabricated electrospun nanofibers of PNCA composition.

### Coating of CNPS at PNCA electrospun nanofibers

A piece of nickel foam with dimensions (1 × 2 cm), which had been covered with electrospun nanofibers made of PNCA, was further modified with CNPS. Briefly, a homogeneous solution of CNPS was meticulously made by dispersing 5 mg of the compound in 1 mL of deionized water, and thereafter subject to ultrasonication for 30 min. Following that, the uniform solution was further applied onto the electrospun nanofibers using the drop-casting technique. To ensure uniform deposition and improved adhesion, the sample was left to dry under ambient conditions for 2 h before undergoing final drying at 37 °C in an oven. The modified electrode, referred to as CNPS@PNCA electrospun nanofibers, was next subjected to drying at a temperature of 37 °C in an oven, thus making it appropriate for electrocatalytic measurements.

## Electronic supplementary material

Below is the link to the electronic supplementary material.

Supplementary Material 1

## Data Availability

The authors declare that the data supporting the findings of this study are available within the paper and its supplementary information files.
