# Supplementary material for: Machine learning assisted Co3O4/NiO popsicle sticks-infused electrospun nanofibers for efficient oxygen evolution reaction
Source: Sci Rep. 2025 Mar 28;15:10677. doi: 10.1038/s41598-025-95130-7 (PMC11950386; doi:10.1038/s41598-025-95130-7)
Supplement: Supplementary file 1 — Supplementary Material 1 [file 41598_2025_95130_MOESM1_ESM.docx]

**Supporting Information**

Machine Learning Assisted Co_3_O_4_/NiO Popsicle Sticks-Infused Electrospun nanofibers for Efficient Oxygen Evolution Reaction

Azza A. Al-Ghamdi^1^, Abdul Sami^2^, Salah M. El-Bahy^3^, Merfat M. Alsabban^1^, Wajid Sajjad^2^, Ahlam I. Al-Sulami^1*^, Muhammad Waseem Fazal^2^, Reema H. Aldahiri^1^, Fatimah Mohammad H. Al-Sulami^1^, Muhammad Ali Khan^2*^, Naeem Akhtar^2*^

^1^University of Jeddah, College of Science, Department of Chemistry, Jeddah 21589, Saudi Arabia

^2^Institute of Chemical Sciences, Bahauddin Zakariya University (BZU), Multan 60800, Pakistan

^3^Department of Chemistry, Turabah University College, Taif University, P.O. Box 11099, Taif 21944, Saudi Arabia

Dr. Naeem Akhtar: [naeemakhtar@bzu.edu.pk](mailto:naeemakhtar@bzu.edu.pk)

Muhammad Ali Khan: malikhan@bzu.edu.pk

Ahlam I. Al-Sulami: 04102237@uj.edu.sa


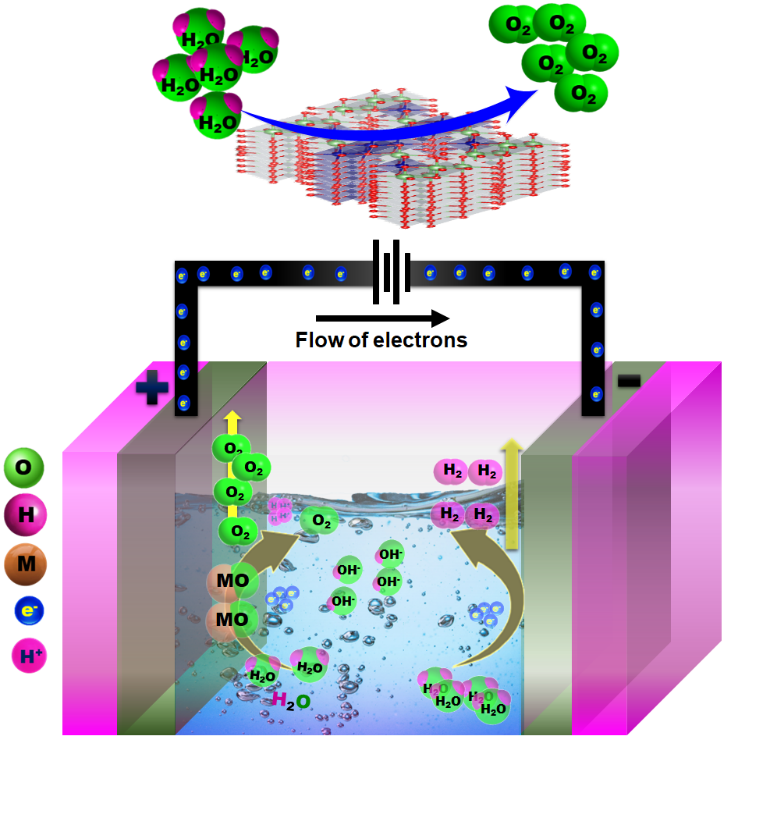


**Scheme 1.** Demonstration of OER Reaction Mechanism at the Surface of CNPS@PNCA based electrode.

# Section 1

**Instrument details**

Instruments used during this scientific work were Fourier transform infrared spectroscopy (FTIR) IRA affinity-1S spectrophotometer-USA to record FTIR spectra of BM-MOF, Co-SA, cellulose acetate, polyaniline and cellulose acetate/polyaniline nanocomposite in the range of 500-4000 cm-1. X-ray diffraction (XRD) was measured by Rigaku, Mini flex-II-Japan by means of Cu Kα (with a scan angle: 5⁰-80⁰, at 40 kV, 40 mA, and (2q). The surface morphological analyses of the synthesized materials were analyzed using scanning electron microscopy (SEM) at a Zeiss Evo 50 XVP equipped with energy dispersive X-ray (EDAX) (Oxford instruments INCA, X.act, S.No. 56756, UK). Raman spectra was obtained using in Via Raman Microscope by RENISHAW UK with excitation laser of 514nm (laser power: 100%, grating: 1800 I/mm) and laser exposure time of 10s. Electrospinning machine (TONG LI TECH CO LTD, China) was used for the formation of nanofibers of PNCA composite. All the electrochemical investigations were carried out using Gamry interface (1010E) Potentio state. Th is Potentio state instrument contains three electrodes such as working electrode, reference electrode and auxiliary electrode. In this work, synthesized electrode CNPS@PNCA was used as working electrode, Ag/AgCl used as reference electrode and platinum wire used as auxiliary electrode.


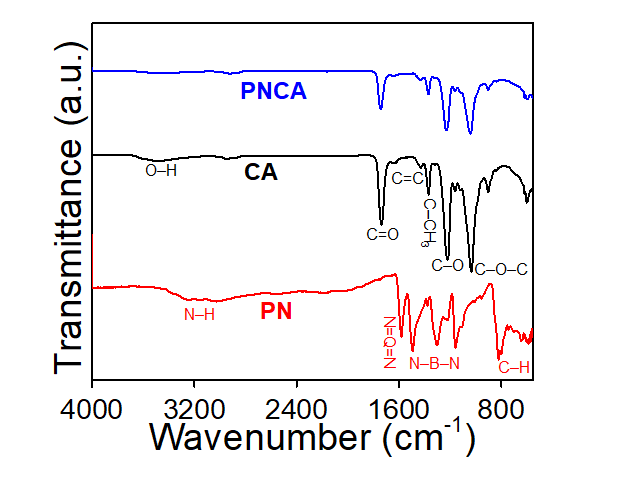


**Figure S1.** FTIR spectra of CA, PN, and PNCA.


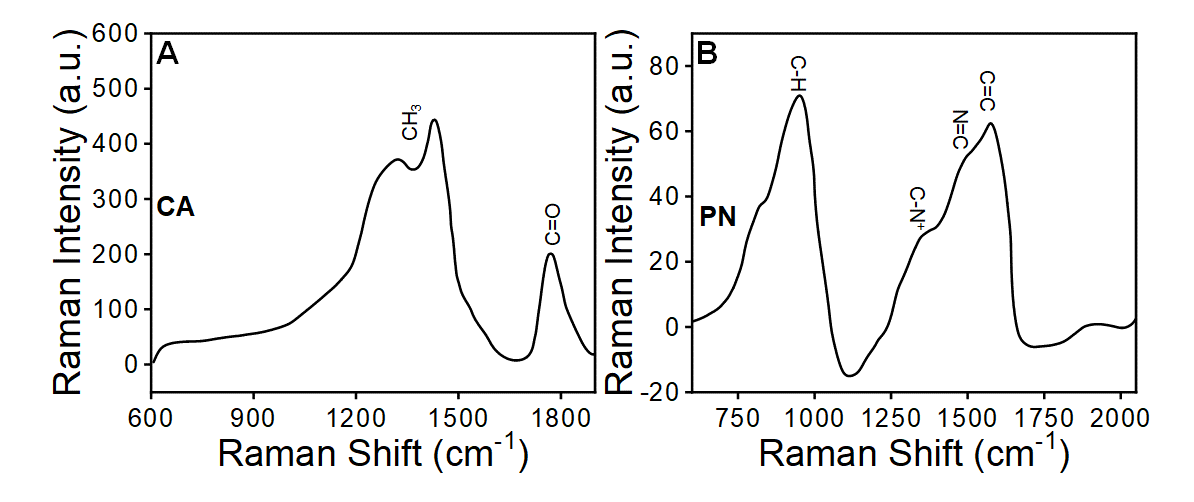


**Figure S2.** Raman spectra of (A) CA and (B) PN.

# Section 2

**ML methods for optimization and evaluation**

The present study delved into examining the impact of ML in predicting overpotential on various compositions of COPS involving implementation of various regression models (LR, ENR, DTR, RFR, GBR, KNNR, SVR and XGBR) on experimental dataset (ED). Multiple regression models, were employed to establish quantitative relationship between a dependent variable (Overpotential) and one or more independent variables (concentration of Ni(NO_3_)_2_·6H_2_O and Co(NO_3_)_2_·6H_2_O in the fabricated COPS). The evaluation of dataset done by regression model primarily centers on the partitioning of available data into three distinct subsets: the training set, the validation set, and the test/prediction set. Initially, the training dataset is employed to train the regression models, followed by rigorous testing against both the validation and test datasets. The performance and accuracy of predictions are meticulously analyzed through the error metrics, specifically RSME and R^2^.

***
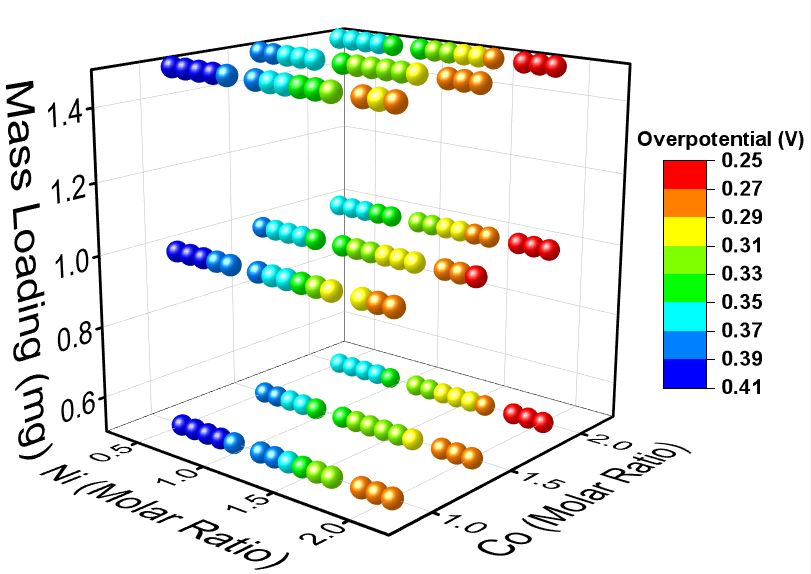
***

**Figure S3.** illustrates the impact of various experimental parameters on the predicted overpotential of the GBR model.

# Section 3

**Electrochemical measurement**

A computer controlled GAMRY Potentio state workstation was used for all electrochemical studies, such as cyclic voltammetry (CV), linear sweep voltammetry (LSV), electrochemical impedance spectroscopy (EIS) and chronoamperometry into water oxidation for the test. Cyclic voltammetry was used to study redox process occurred at the electrode/electrolyte interface. The electrochemical impedance spectroscopy approach was used to measure the electrical resistance of a particular analyte.

The electrochemical experiments were performed by using standard three-electrode system which was enclosed in Teflon cell. In order to clean the electrochemical cell, it was washed by a mixture solution containing 3:1 of HNO_3_ and H_2_SO_4_ and then with ultrapure distilled water. Moreover, it was rinsed numerous times first with ultrapure distilled water and then with acetone. After washing, it was dried in an oven for 30–40 minutes at 80°C. Before placing the counter electrode, which was Pt wire, into electrochemical cell, it was washed with ultrapure water and dipped in a 20% solution of HNO_3_ for a few minutes. Commercially available nickel foam (NF) treated with CNPS@PNCA and saturated silver–silver chloride (Ag|AgCl) electrode was used as working and reference electrode, respectively. At room temperature, all measurements were performed in a 1 M KOH electrolyte solution of pH of 13.5. At scan rates of 5 to 25 mV s^-1^, CVs were determined by intentionally cycling from a positive to a negative potential. The solution of electrolyte was purified with argon gas for 30 minutes before measuring the electrochemical activity. The working electrode's geometrical area was taken into account when calculating all current values. All potentials were recorded after 50 percent IR adjustment, which was done manually using the formula:

$$Eactual=Eexperimental-IR (S1)$$

The following equation was used to convert all potentials collected vs. Ag/AgCl into RHE potentials:

$$ERHE=E Ag/AgCl+0.059 pH+EO Ag/AgCl (S2)$$

Here, E Ag/AgCl represents the measured potential against the Ag/AgCl electrode, E_0_ Ag/AgCl represents the typical thermodynamic potential (0.197 V) of Ag/AgCl, and E_RHE_ represents the estimated potential vs. RHE.

## Tafel Slope calculations from the polarization curve of CNPS@PNCA

To evaluate the kinetics and catalytic performance, Tafel plot was plotted between over potential and log of current density in the linear portion of steady state polarization curve. It can be described using the equation.

$$ŋ=a+\left( 2.303\frac{RT}{ꭤnF} \right)*\log j (S3)$$

Here, over potential is represented by ŋ, charge transfer coefficient byꭤ, the number of electrons take part in reaction by n, current density by j, faraday constant by F. The 2.303RT/anF value refers to the slope.

**Electrochemically active surface area of CNPS@PNCA**

Evaluating the electrochemical double-layer capacitance and performing CV measurements at various scan ranging from 5-25 mV/s, the electrochemically active surface area of the catalyst was measured. By fitting the average current density vs scan rate curve, the electrochemical capacitance may be calculated easily. The CV was performed within non-faradic region. The electrochemical active area was calculated by adopting already reported method [S[1]] and is given below

*C_dl_* = *slope*/2 (S4)

Electro-active surface area = *C_dl_/C_sp_* (S5)

**Calculation for electrochemical active area of NC-Co-SAC@CP/NF**

Straight-line equation derives from “Figure S3B” is shown in

J = 0.013x + 0.029 R² = 0.9985 (S6)

*C_dl_* = *slope*/2 = 13/2 = 6.5 mF cm^-2^

Slope = linear fit between the scan rate vs current density

Electro-active surface area = *C_dl_/C_sp_*

= 6.5/0.04 = 162.5 cm^2^

**OH^−^ ion adsorption capability of the developed electrodes**

Laviron equation is given below

$$Ec=E\frac{1}{2}-\left( \frac{RT}{\alpha nF} \right)*\ln\left( \frac{\alpha nF}{RTks} \right)-\left( \frac{RT}{\alpha nF} \right)*\ln\left( v \right)(S7)$$

Where, Ec and E1/2 are the reduction and formal potential of metal redox T, F, ks and R, stand for absolute temperature, Faraday constant, redox constant, and general gas constant, respectively. Whereas n and α represent the number of electrons transferred and electron transfer coefficient.

**
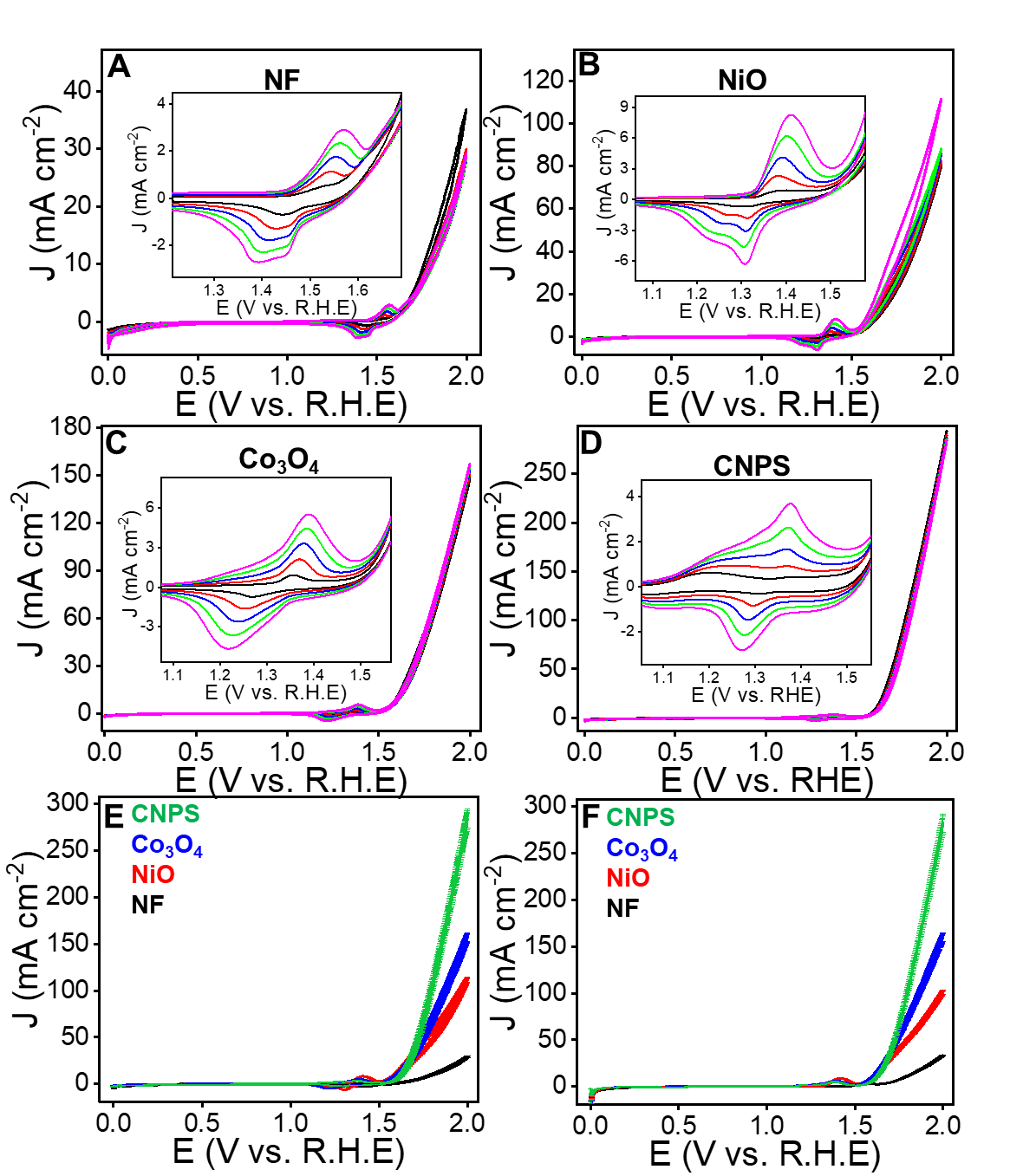
**

**Figure S4.** A-D illustrates CV of NF, NiO, Co₃O₄, and CNPS. E-F Comparative CV and LSV curves of NF, NiO, Co₃O₄, and CNPS at a 25 mV/s scan rate, with error bars representing standard deviations from three repetitions.


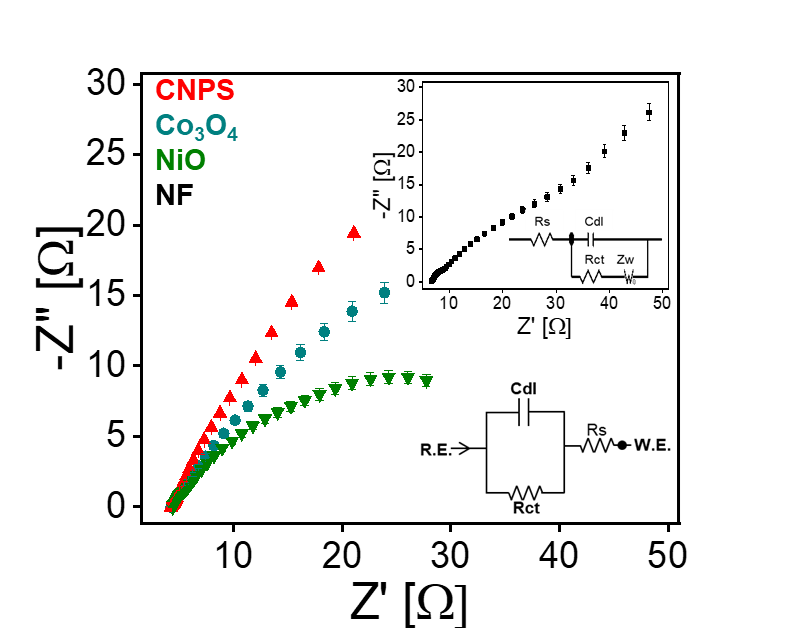


**Figure S5.** Nyquist plot with the Randle’s circuit, representing the solution resistance and charge transfer resistance at the electrode–electrolyte interface.


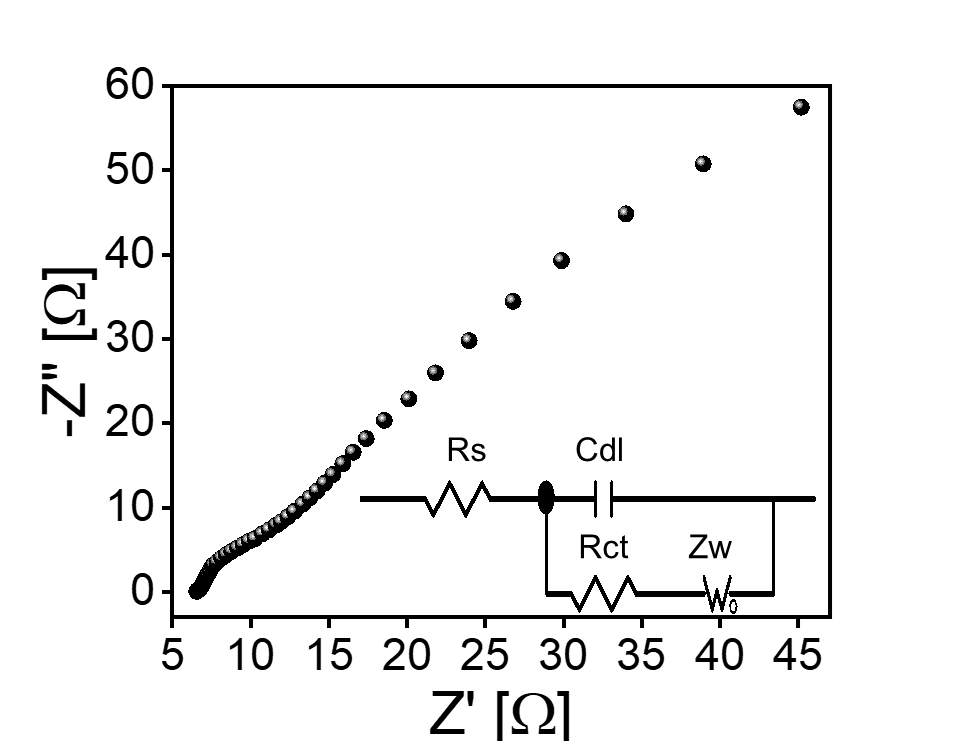


**Figure S6.** Nyquist plot with the Randle’s circuit, representing the solution resistance and charge transfer resistance at the electrode–electrolyte interface of NF.


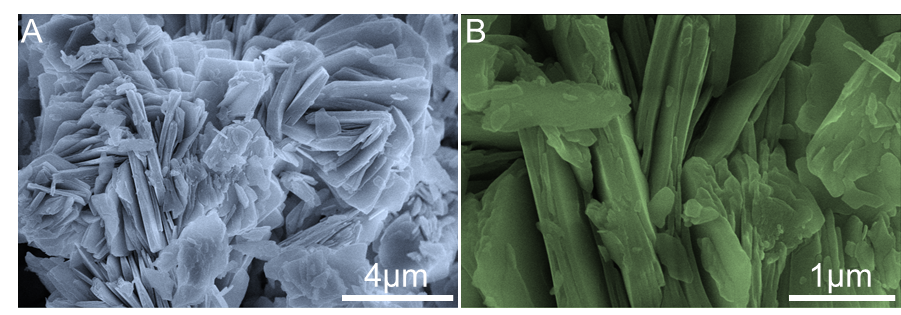


**Figure S7.** SEM images of CNPS (A) low and (B) high magnified.

**Table S1. represents the RMSE, and R^2^ of each ML algorithm on training, validation, and test dataset**

| **Model** | **Train RMSE** | **Validation RMSE** | **Test RMSE** | **R^2^ Score** |
| --- | --- | --- | --- | --- |
| LR | 0.0071 | 0.0080 | 0.0085 | 0.9623 |
| RR | 0.0075 | 0.0084 | 0.0101 | 0.9469 |
| DTR | 0 | 0.0051 | 0.0057 | 0.98291 |
| GBR | 0.0009 | 0.0023 | 0.0030 | 0.9953 |
| KNNR | 0.0098 | 0.0134 | 0.0135 | 0.9060 |
| SVR | 0.0386 | 0.0411 | 0.0448 | -0.0281 |

**Table S2. Comparison table of Electroactive Surface Area (cm^2^) with already reported electrode**

| Sr. No | Electrode | Electroactive Surface Area (cm^2^) |
| --- | --- | --- |
| 1 | NiCoP/NF | 122.5 |
| 2 | NC-Co-SAC/NF | 133.75 |
| 3 | NiCo@NC | 23 |
| 4 | NiCo@NCNTs | 185.75 |
| 5 | NiO/Co_3_O_4_ | 125.25 |
| 6 | MgCo_2_O_4_ | 206.25 |
| 7 | Co_9_S_8_@NOSC-900 | 245 |
| 8 | CNPS@PNCA | 162.5 |

**Table S3. Comparison table of Tafel slope with already reported electrode**

| Sr. No | Electrode | Tafel Slope (mV/dec) | Overpotential (mV) | Electrolyte | Reference |
| --- | --- | --- | --- | --- | --- |
| 1 | NiCoP/NF | 87 | 280 | 1.0 M KOH | S[2] |
| 2 | Co_9_S_8_@NOSC-900 | 68 | 340 | 1.0 M KOH | S[3] |
| 3 | NC-Co-SAC/NF | 72 | 340 | 1.0 M KOH | S[4] |
| 4 | MgCo2O4 | 90 | 278 | 1.0 M KOH | S[5] |
| 5 | NiCo@NC | 98 | 539 | 1.0 M KOH | S[6] |
| 6 | NiO/Co_3_O_4_ | 63 | 330 | 1.0 M KOH | S[7] |
| 7 | NiCo@NCNTs | 177 | 420 | 1.0 M KOH | S[8] |
| 8 | CNPS | 75.4 | 249 | 1.0 M KOH | This work |
| 9 | CNPS@PNCA | 62.1 | 237 | 1.0 M KOH | This work |

1 Nickle Copper Phosphide supported on Nickle foam, 4 magnesium/cobalt spinel oxide nanocomposite, 5 Nickle Cobalt alloy nanoparticles decorated on N-doped carbon nanofibers, 7 nitrogen-doped carbon nanotubes encapsulating nickel cobalt alloys, 15 Nickle Cobalt nanochain alloys.

**Reference:**

1. Manzoor, S., et al., *Energy conversion performance of porous ZrTe hybrid derived from chemical transformation of Zr (OH) 4.* 2022. **328**: p. 125264.

2. Liang, H., et al., *Plasma-Assisted Synthesis of NiCoP for Efficient Overall Water Splitting.* Nano Letters, 2016. **16**(12): p. 7718-7725.

3. Huang, S., et al., *N‐, O‐, and S‐tridoped carbon‐encapsulated Co9S8 nanomaterials: efficient bifunctional electrocatalysts for overall water splitting.* 2017. **27**(17): p. 1606585.

4. El-Shishtawy, R.M., et al., *N-Coordinated cobalt single atom-integrated electrospun nanofibers for an efficient oxygen evolution reaction.* New Journal of Chemistry, 2023. **47**(30): p. 14177-14184.

5. Karami, A.M., et al., *Fabrication of PANI supported magnesium/cobalt spinel ternary oxide as an efficient oxygen evolution reaction and removal of organic pollutant.* Ceramics International, 2023.

6. Fu, Y., et al., *NiCo alloy nanoparticles decorated on N‐doped carbon nanofibers as highly active and durable oxygen electrocatalyst.* Advanced Functional Materials, 2018. **28**(9): p. 1705094.

7. Nam, D. and J. Kim, *Development of NiO/Co3O4 nanohybrids catalyst with oxygen vacancy for oxygen evolution reaction enhancement in alkaline solution.* International Journal of Hydrogen Energy, 2022. **47**(38): p. 16900-16907.

8. Yu, J., et al., *Facile synthesis of nitrogen-doped carbon nanotubes encapsulating nickel cobalt alloys 3D networks for oxygen evolution reaction in an alkaline solution.* Journal of Power Sources, 2017. **338**: p. 26-33.
